# Assessment of risk factors of treatment discontinuation among patients on paliperidone palmitate and risperidone microspheres in France, Germany and Belgium

**DOI:** 10.1186/s12888-022-03914-2

**Published:** 2022-06-07

**Authors:** Rui Cai, Flore Decuypere, Pierre Chevalier, Martin Desseilles, Martin Lambert, Eric Fakra, Antonie Wimmer, Pascal Guillon, Stefan Pype, Annabelle Godet, Valeria Borgmeier

**Affiliations:** 1 Real World Evidence, IQVIA, Zaventem, Belgium; 2 Analytics Solutions, IQVIA, Zaventem, Belgium; 3 Corporate Village,, Davos Building, Da Vincilaan 7, 1930 Zaventem, Belgium; 4 Université de Namur, Namur, Belgium; 5 Universitätsklinikum Hamburg-Eppendorf, Hamburg, Germany; 6 CHU de Saint-Etienne, Saint-Etienne, France; 7 Janssen-Cilag, Neuss, Germany; 8 Janssen-Cilag, Issy-les-Moulineaux, France; 9 Janssen-Cilag, Beerse, Belgium

**Keywords:** Antipsychotics, PP1M, PP3M, Risperidone microsphere, Treatment continuation

## Abstract

**Background:**

Long-acting antipsychotics (e.g. 1-monthly (PP1M) / 3-monthly (PP3M) injection forms of paliperidone palmitate) have been developed to improve treatment continuation in schizophrenia patients. We aim to assess risk factors of treatment discontinuation of patients on paliperidone palmitate and risperidone microsphere. Additionally, treatment discontinuation between patients with PP1M and PP3M was compared.

**Methods:**

The IQVIA Longitudinal Prescription databases were used. Risk factors of treatment discontinuation were identified by a multilevel survival regression using Cox proportional hazards model. Kaplan Meier analyses were performed by identified significant risk factors.

**Results:**

Twenty-five thousand three hundred sixty-one patients (France: 9,720; Germany: 14,461; Belgium: 1,180) were included. Over a one-year follow-up period, a significant lower treatment discontinuation was observed for patients newly initiated on paliperidone palmitate (53.8%) than those on risperidone microspheres (85.4%). Additionally, a significantly lower treatment discontinuation was found for ‘stable’ PP3M patients (19.2%) than ‘stable’ PP1M patients (37.1%). Patients were more likely to discontinue when drugs were prescribed by GP only (HR = 1.68, *p* < 0.001 vs. psychiatrist only) or if they were female (HR = 1.07, *p* < 0.001), whereas discontinuation decreased with age (31–50 years: HR = 0.95, p = 0.006 and > 50 years: HR = 0.91, *p* < 0.001 vs. 18–30 years).

**Conclusions:**

This study demonstrates that patients stay significantly longer on treatment when initiated on paliperidone palmitate as compared to risperidone microspheres. It also indicated a higher treatment continuation of PP3M over PP1M. Treatment continuation is likely to be improved by empowering GPs with mental health knowledge and managing patients by a collaborative primary care-mental health model. Further research is needed to understand why females and younger patients have more treatment discontinuation.

**Supplementary Information:**

The online version contains supplementary material available at 10.1186/s12888-022-03914-2.

## Background

Schizophrenia is a remitting psychiatric disorder characterized by significant impairments of mental and social functioning [1]. It is among the most disabling medical disorders and represents a significant economic burden [2]. The prevalence of schizophrenia is about 0.5% in Europe [3]. As a chronic disorder, schizophrenia can be effectively controlled but likely requires lifelong treatment, even when symptoms have subsided [4]. Antipsychotic medications (APs) play a central role in recommendations related to the treatment as they can control symptoms and improve the outcomes of schizophrenia [5]. However, discontinuation of APs remains an issue and is associated with high risks of relapse, rehospitalization and increased disease severity and medical resource use [6–10].

Long-acting antipsychotic treatment (LAT) offers an important alternative to oral APs in the context of medication continuation. Several antipsychotics, such as risperidone, olanzapine, paliperidone palmitate, and aripiprazole, are approved in Europe in both oral and LAT preparations. Reported treatment continuation differ markedly depending on the study design [11]. In randomized controlled trials (RCT) or prospective observational studies, adherence is optimized by protocol and is a major driver of treatment continuation [12]. Therefore, a retrospective design is recommended when studying questions related to effectiveness, as looking back to what already happened is by its nature noninterventional [13, 14]. Moreover, by using nationwide electronic databases, a large number of patients can be included in retrospective observational studies to achieve enough statistical power and facilitate the generalization of findings [15].

Observational studies have shown treatment continuation increased with the use of LAT compared to oral APs [15, 16]. However, difference in terms of treatment continuation between LATs was not very well studied. Recent studies have shown paliperidone palmitate can result in similar or better treatment continuation as compared to aripiprazole [17] and other LAT such as risperidone microspheres [18–20]. However, factors associated with treatment continuation were not explored in these studies. Furthermore, a 3-monthly injection form of paliperidone palmitate (PP3M) was approved for market authorization in Europe in 2016. PP3M is indicated for patients who are adequately treated with 1-monthly paliperidone palmitate injectable (PP1M), and do not require dose adjustment. Treatment continuation of patients treated with PP3M is supposed to be superior to other LAT, however, has not yet been proved by real-world data. The primary aim of our study is to assess risk factors of treatment discontinuation of patients newly initiated on paliperidone palmitate and risperidone microsphere. The secondary aim is to compare treatment continuation between patients with PP1M and PP3M.

## Methods and materials

### Data sources

Data were extracted from the IQVIA Longitudinal Prescription databases (LRx) for France, Germany and Belgium and have covered approximately 33%, 60% and 25% respectively of all retail pharmacies. The three countries have been selected as data were collected in a similar way. Moreover, the three countries are among the countries with the highest number of patients initiated on PP3M as of its commercial availability in Europe. The databases contain actual prescription pickup data from pharmacy records for anonymized patients. Only pharmaceutical products purchased in retail pharmacies are recorded and no data from the hospital pharmacies are captured. Data from retail pharmacies were further de-identified by a Trusted Third Party before transferring to IQVIA. Key patient information includes prescribed and dispensed drug, molecule, brand and generic name, manufacturer, form, strength, dose, pack size, method of administration, quantity, and prescription dispensing date. Other data include gender, physician specialty, concomitant medication, cost of prescriptions, and health insurance status. Age was available in the German and French but not the Belgian database. Information on patients’ diagnosis and disease severity was not available. These databases have been used by IQVIA to study questions of persistence in schizophrenia [19] as well as in other psychiatric [21] and persistence topics [22, 23]. A unique patient identifier was used to ensure patients visiting different pharmacies within the panel were followed in all the three countries. Only data from pharmacies which transmitted data every month were used.

Ethical approval was not required for this research as only deidentified/anonymized electronic databases were used. It was therefore not necessary to acquire any administrative permissions and/or licenses to access clinical/personal data used in this research.

### Patient selection

**Fig. 1:**
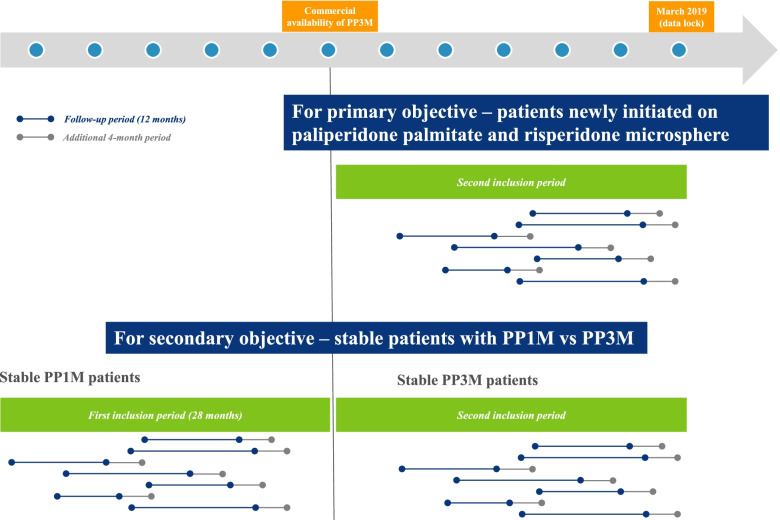
Patient selection process

Two inclusion periods were defined (as shown in Fig. 1). To ensure enough patients could be followed up for 16 months, we defined the first inclusion period as between 28 and one months prior to the commercial availability of PP3M. The 16 months consisted of 12-month follow up and an additional four-month period to avoid artificial discontinuation (i.e. erroneous characterization of censored patients as having discontinued). The second inclusion period was defined as between the month of the commercial availability of PP3M and March 2019 (data lock). The length of the second inclusion period was different across the three countries as PP3M became commercially available at different times (February 2017 in France, June 2016 in Germany and November 2016 in Belgium). All patients in the second inclusion period were followed for 12 months as well.

For the primary objective, patients newly initiated on paliperidone palmitate and risperidone microsphere during the second inclusion period were selected. Patients newly initiated were those who had not purchased the same drug in the 12 months preceding the index treatment. For the secondary objective, “stable” PP3M patients in the second inclusion period and “stable” PP1M patients in the first inclusion period were selected. Stable patients refer to patients who had purchased a minimum number of packs of the study drug within six months preceding the index treatment. Per PP3M label, patients need to show stability on PP1M for a minimum of four months before switching to PP3M. To be comparable with PP1M patients, PP3M patients for whom the stabilization can be observed were selected. The minimum number of packs was defined to cover four months of treatment. Moreover, as treatment continuation with PP1M was impacted artificially following the availability of PP3M– as patients may discontinue PP1M simply to switch to PP3M—patients on PP1M prior to the availability of PP3M were selected.

Patient inclusion criteria were: (1) had at least one transaction of any drug during six months prior to initiation of the study drugs; (2) had at least two transactions of any drug on two distinct dates during the 12 months after initiation; (3) 18 years old and above at index date as the study drugs were not indicated for patients younger than 18 years. Age was not available in the Belgian database, thus this inclusion criteria was not applied; (4) patients with no missing or unknown values of studying variables, except for patients with missing values of age in Belgium; (5) patients not purchasing two or more LATs concomitantly.

### Treatment continuation

A patient was considered as being continuously on treatment either until the treatment was stopped or interrupted for a period longer than a pre-defined permissible gap. The permissible gap is the maximum time (following the previous prescription date) in which a patient should have revisited the pharmacy to be considered as continuing the treatment. The permissible gap was defined as:

1 $$\left[\mathrm{coverage}\;\mathrm{period}\;\mathrm{of}\;1\;\mathrm{unit}\right]\times\left[\mathrm{number}\;\mathrm{of}\;\mathrm{units}\;\mathrm{in}\;\mathrm{the}\;\mathrm{last}\;\mathrm{purchase}\right]+\left[\mathrm{grace}\;\mathrm{period}\right]$$

The time to treatment discontinuation was calculated as:

2 $$\begin{array}{c}\left[\mathrm{Time}\;\mathrm{between}\;\mathrm{initiation}\;\mathrm{and}\;\mathrm{last}\;\mathrm{purchase}\;\mathrm{before}\;\mathrm{treatment}\;\mathrm{stop}\right]\\+\left[\mathrm{coverage}\;\mathrm{period}\;\mathrm{of}\;1\mathrm{unit}\right]\times\left[\mathrm{number}\;\mathrm{of}\;\mathrm{units}\;\mathrm{in}\;\mathrm{the}\;\mathrm{last}\;\mathrm{purchase}\right]\end{array}$$

The coverage period was based on the summary of product characteristics (SmPC) by the European Medicines Agency. The grace period reflects the additional time allowed beyond the coverage period for deviations from the theoretical prescription frequency. In the base case, the grace period was set as the coverage period of 1 unit plus 150% of the administration window to avoid a missed dose. It was calculated as such so that if a patient purchases one-unit drug from pharmacies not included in the databases, the patient would still be considered on continuous treatment. The administration window of risperidone microspheres was not mentioned in the SmPC and was set as half of that of PP1M. A sensitivity analysis was conducted across products, applying an equal grace period of 120 days (proxy to the longest grace period in the base case). The coverage and grace period lengths were summarized in Table 1.

**Table 1:** Coverage period and grace period definitions (in days)

	**PP3M**	**PP1M**	**Risperidone microspheres**
Coverage period	90	30	14
Grace period (base case)^a^	90 + 21 = 111	30 + 10 = 40	14 + 5 = 19
Grace period (sensitivity analysis)	120	120	120

^a^The grace period in the base case was set as the coverage period of 1 unit plus 150% of the administration window to avoid a missed dose. The coverage period and administration window was based on the summary of product characteristics (SmPC) by the European Medicines Agency. The administration window of risperidone microspheres was not mentioned in the SmPC and was set as half of that of PP1M

### Data extracted

Of the selected patients, data about gender, any product purchased between six months before and three months after the index date, date of transactions, dosage, and specialty of the prescribers of the study drugs were extracted from the three databases and pooled together. Age was extracted from the French and German databases only, as it was not available in Belgium.

Previous treatments, i.e. products purchased six months before the index date were classified as index drug; other LATs; oral APs; and no AP. Patients with no purchase of other APs during the first three months after index, except for oral risperidone or oral paliperidone, were referred as patients on monotherapy. Else patients were referred to as patients on combination therapy. The recommended monthly dose of the three study drugs was similar as per the SmPC (50 mg). Patients were grouped as patients who had an average dosage of < 75, 75–125, and > 125 mg / 30 days. Specialty of prescribers were grouped as psychiatrist only, general practitioner (GP) only, psychiatrists and GP, and other specialties. In France and Germany, prescriptions at hospitals but dispensed in pharmacies do not contain information on specialty of prescribers. In those cases, prescriptions were assumed to originate from a psychiatrist.

### Statistical analysis

For primary objective – evaluation of the risk factors of treatment discontinuation of patients newly initiated on paliperidone palmitate and risperidone microspheres.

#### Patient characteristics

Patient characteristics such as gender, age, previous treatment, and speciality of the prescribers were used as categorical variables and presented as numbers (percent) for all patients and by country.

#### Risk factors of treatment discontinuation

A multilevel survival regression using Cox proportional hazards regression model with mixed effects (frailty model) was used to identify risk factors of treatment discontinuation. The level one variables were gender, age, study drug, previous treatment, number of distinct molecules purchased, dosage, combination therapy, and specialty of the prescribers. Country was used as a clustering level two variable. All variables were tested with univariate analyses and were incorporated in the multivariate analysis when the *p*-value was < 0.20. Final risk factors were selected through stepwise selection. Hazard ratios for the risk factors were calculated. In case age had a *p*-value < 0.20 in the univariate analysis, two sets of multivariate analyses were run, i.e. one with (excluding the Belgian data) and one without age (including Belgian data) as a covariate.

#### Kaplan Meier analyses for the time to treatment discontinuation

To determine median time from treatment initiation to discontinuation and the percentage of patients discontinuing the study drugs during the 12-month follow up, Kaplan Meier survival analyses were performed. The analyses were performed by significant risk factors identified from multivariate Cox regression. Sensitivity analyses of survival analyses using a different grace period were performed.

For secondary objective – comparison of treatment continuation between stable patients with PP3M and PP1M.

A similar set of analyses about patient characteristics and risk factors of treatment discontinuation were performed to compare the treatment continuation between patients with PP3M and PP1M. To visualize the difference of time to discontinuation between patients with PP1M and PP3M, a Kaplan Meier survival analysis was performed.

The Statistical Analysis System SAS (SAS for XP PRO, Release 9.4 TS2 M3; SAS Institute Inc., Cary, NC, USA) was used to perform analyses. For all statistical tests, *p* < 0.05 was considered significant.

## Results

For primary objective – evaluation of the risk factors of treatment discontinuation of patients newly initiated on paliperidone palmitate and risperidone microspheres.

### Patient characteristics

In total, 25,361 patients (9,720 in France; 14,461 in Germany and 1,180 patients in Belgium) were included in the final analysis. Table 2 shows the characteristics of these patients. Approximately 61% of all patients were men, and 71% were patients newly initiated on paliperidone palmitate.

**Table 2:** Characteristics of patients newly initiated on paliperidone palmitate and risperidone microsphere

	**Total**	**France**	**Germany**	**Belgium**
*Total*	25,361 (100%)	9,720 (100%)	14,461 (100%)	1,180 (100%)
*Gender*				
Women	10,014 (39%)	3,472 (36%)	6,022 (42%)	520 (44%)
Men	15,347 (61%)	6,248 (64%)	8,439 (58%)	660 (56%)
*Age*				
18–30 years	4,687 (18%)	2,236 (23%)	2,451 (17%)	-
31–50 years	11,348 (45%)	4,906 (50%)	6,442 (45%)	-
> 50 years	8,146 (32%)	2,578 (27%)	5,568 (39%)	-
*Type of treatment*				
Paliperidone palmitate	18,028 (71%)	7,463 (77%)	9,756 (67%)	809 (69%)
Risperidone microspheres	7,333 (29%)	2,257 (23%)	4,705 (33%)	371 (31%)
*Previous treatment*				
Other LAT	4,658 (18%)	1,807 (19%)	2,736 (19%)	115 (10%)
Oral AP	9,456 (37%)	2,902 (30%)	6,146 (43%)	408 (35%)
No AP	11,247 (44%)	5,011 (52%)	5,579 (39%)	657 (56%)
*Average dosage per 30 days*^*a*^				
< 75 mg	5,000 (20%)	1,787 (18%)	2,889 (20%)	324 (27%)
75–125 mg	13,208 (52%)	4,708 (48%)	7,968 (55%)	532 (45%)
> 125 mg	7,153 (28%)	3,225 (33%)	3,604 (25%)	324 (27%)
*Combination therapy*				
Monotherapy	12,480 (49%)	5,574 (57%)	6,252 (43%)	654 (55%)
Combination therapy	12,881 (51%)	4,146 (43%)	8,209 (57%)	526 (45%)
*Specialty of the prescribers*				
GP only	1,867 (7%)	622 (6%)	831 (6%)	414 (35%)
Psychiatrist + GP	2,736 (11%)	1,074 (11%)	1,310 (9%)	352 (30%)
Psychiatrist only	19,492 (77%)	7,485 (77%)	11,634 (80%)	373 (32%)
Other	1,266 (5%)	539 (6%)	686 (5%)	41 (3%)
*Other treatments*^b^				
None	6,676 (26%)	1,220 (13%)	5,267 (36%)	189 (16%)
1 molecule	3,612 (14%)	1,128 (12%)	2,292 (16%)	192 (16%)
2 molecules	3,167 (12%)	1,193 (12%)	1,778 (12%)	196 (17%)
> 2 molecules	11,906 (47%)	6,179 (64%)	5,124 (35%)	603 (51%)

^a^The recommended monthly dose of the three study drugs was similar per SmPC (50 mg per 30 days), i.e. risperidone microspheres for 25 mg every two weeks, PP1M for 50 mg per month and PP3M for 175 mg per three months

^b^Based on the number of distinct molecules other than antipsychotics purchased, patients were classified into four groups: patients who purchased 0, 1, 2 and more than 2 distinct other molecules during the first three months after index

### Risk factors of treatment discontinuation

Association between treatment discontinuation and possible risk factors were expressed as hazard ratio (HR). A hazard ratio greater than 1 suggests an increased risk, and a hazard ratio below 1 suggests a smaller risk. Univariate Cox regression analyses revealed that patients newly initiated on risperidone microsphere were more than twice as likely (vs. paliperidone palmitate, hazard ratio (HR) = 2.08, *p* < 0.001) to discontinue treatment. The following subgroups were more likely to discontinue treatment: females (HR = 1.07, *p* < 0.001), patients previously treated with oral AP (HR = 1.39, *p* < 0.001) or no treatment (HR = 1.36, *p* < 0.001), patients receiving combination therapy (HR = 1.05, *P* = 0.003), and patient receiving drugs prescribed by general practitioner (GP) only (HR = 1.68, *p* < 0.001) (Table 3). On the other hand, patients receiving a relatively high dose of treatment (HR = 0.94, *p* < 0.003 for patients receiving an average dose of 75-125 mg and HR = 0.81, *p* < 0.001 for patients receiving more than 125 mg per 30 days) and patients receiving drugs prescribed by psychiatrists and GPs (HR = 0.81, *p* < 0.001) or by other specialists (HR = 0.82, *p* < 0.001) were less likely to discontinue treatment.

**Table 3:** Risk factors of treatment discontinuation of paliperidone palmitate or risperidone microsphere by univariate and multivariate Cox regression analysis

			**Univariate**		**Multivariate (including Belgian data)**		**Multivariate (excluding Belgian data)**	
Variable	Reference	Class	HR	*P* value^	HR	*P* value^	HR	*P* value^
Gender	Men	Women	1.07	** < .0001**	1.05	**0.001**	1.05	**0.002**
Age^a^	18–30 years	31–50 years	0.96	0.051	-	-	0.95	**0.006**
		> 50 years	0.97	0.20	-	-	0.91	** < .0001**
Type of treatment	Paliperidone palmitate	Risperidone microsphere	2.08	** < .0001**	1.96	** < .0001**	1.97	** < .0001**
Previous treatment	Other LAT	Oral AP	1.39	** < .0001**	1.22	** < .0001**	1.22	** < .0001**
		No AP	1.36	** < .0001**	1.22	** < .0001**	1.21	** < .0001**
Dosage per 30 days^b^	< 75 mg	75–125 mg	0.94	**0.003**	0.98	0.36	0.98	0.27
		> 125 mg	0.81	** < .0001**	0.95	**0.018**	0.95	**0.037**
Combination therapy	Monotherapy	Combination therapy	1.05	**0.003**	1.05	**0.001**	1.06	**0.001**
Specialty of the prescribers	Psychiatrist only	GP only	1.68	** < .0001**	1.37	** < .0001**	1.36	** < .0001**
		Psychiatrist + GP	0.81	** < .0001**	0.76	** < .0001**	0.78	** < .0001**
		Other	0.82	** < .0001**	0.82	** < .0001**	0.81	** < .0001**
Other treatments^c^	None	1 molecule	1.04	0.11	1.02	0.46	1.01	0.63
		2 molecules	1.03	0.23	0.98	0.47	0.98	0.53
		> 2 molecules	1.05	**0.008**	0.99	0.77	1.00	0.85

^*P*-values in bold are those with *p*-values < 0.05

^a^Age was not available in the Belgian database. The Belgian data was not included in the univariate analysis using age as a covariate but was used all remaining univariate analyses

^b^The recommended monthly dose of the three study drugs was similar per SmPC (50 mg per 30 days), i.e. risperidone microspheres for 25 mg every two weeks, PP1M for 50 mg per month and PP3M for 175 mg per three months

^c^Based on the number of distinct molecules other than antipsychotics purchased, patients were classified into four groups: patients who purchased 0, 1, 2 and more than 2 distinct other molecules during the first three months after

In the multivariate Cox regression analysis, the adjusted HR was similar as in the univariate analysis. The difference in treatment discontinuation between patients receiving an average dose of > 125 mg and those receiving less than 75 mg per 30 days remained significant. However, the difference in treatment discontinuation between patients receiving an average dose of 75-125 mg and those receiving less than 75 mg per 30 days was no longer significant suggesting that dose as such might not be an independent risk factor. When age was included in the multivariate Cox regression using French and German data, results show that older patients; 31–50 years (HR = 0.95, *p* = 0.006) and > 50 years (HR = 0.91, *p* < 0.001) were less likely to discontinue treatment (Table 3).

### Survival analyses of treatment discontinuation

**Fig. 2:**
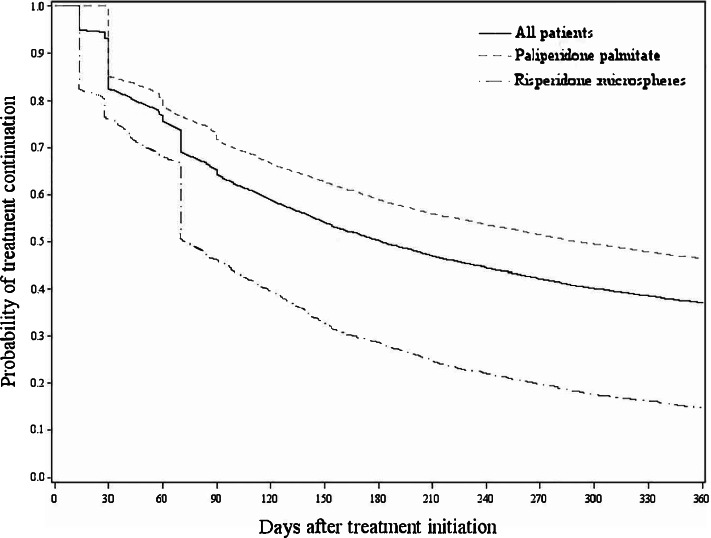
Overall and by product treatment discontinuation (total sample) – patients newly initiated on paliperidone palmitate and risperidone microspheres

**Fig. 3:**
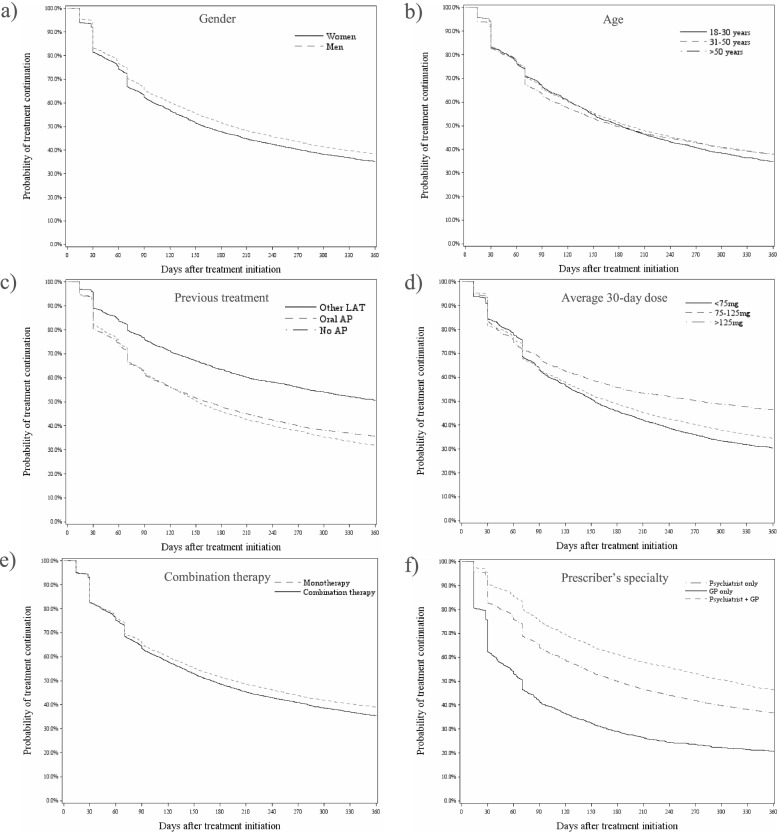
Treatment discontinuation by risk factors (total sample) – patients newly initiated on paliperidone palmitate and risperidone microspheres

Figure 2 shows the overall and by product Kaplan–Meier curve for the time from treatment initiation until discontinuation. The overall median time from treatment initiation until discontinuation was 181 (95% CI: 176—186) days. Median time from risperidone microsphere initiation until treatment discontinuation (72 [95% CI: 70—78] days) was significantly shorter compared to paliperidone palmitate (289 [95% CI: 280—305] days). Observed discontinuation rates were 53.8% and 85.4% among patients on paliperidone palmitate and risperidone microspheres respectively after one-year follow up. The difference in treatment discontinuation between paliperidone palmitate and risperidone microspheres remains in the sensitivity analysis when using an equal grace period of 120 days for all study drugs. Figure 3 shows the Kaplan–Meier curve for the time from treatment initiation until discontinuation by significant risk factors. The per country Kaplan–Meier curves by significant factors were shown in Figure S2- Figure S8.

For secondary objective – comparison of treatment discontinuation between stable patients with PP3M and PP1M.

**Fig. 4:**
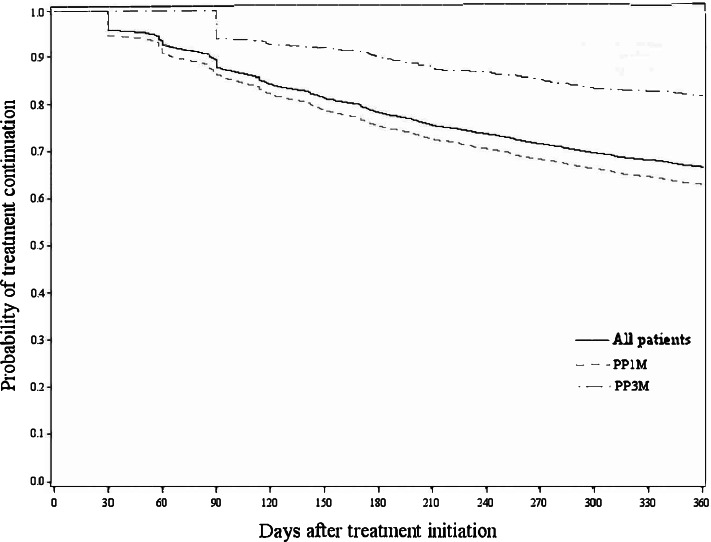
Overall and by product treatment discontinuation – stable PP3M patients vs. stable PP1M patients

Table S1 shows the characteristics of stable PP1M and PP3M patients. Approximately 37% of all patients were women, and 80% were stable PP1M patients. A similar set of risk factors of treatment discontinuation as those for the primary objective were identified (Table S2). The discontinuation rates of stable PP3M patients (19.2%) were significantly lower than stable PP1M patients (37.1%) after one-year follow up (*p* < 0.001, Fig. 4). The difference in treatment discontinuation between PP3M and PP1M remains significant (*p* < 0.001) in a sensitivity analysis using an equal grace period of 120 days for all study drugs (Figure S9).

## Discussion

This study shows that patients newly initiated on paliperidone palmitate had a significantly higher treatment continuation compared to those on risperidone microspheres based on national, longitudinal, prescription databases of retail pharmacies in France, Germany and Belgium. Receiving drugs prescribed by GP only, being previously treated with oral AP or no treatment, and receiving combination therapy were risk factors for treatment discontinuation. On the other hand, older patients and patients receiving relatively high dose of treatment were less likely to discontinue treatment. Additionally, stable PP3M patients had a significantly higher treatment continuation than stable PP1M patients.

Results from our study are in line with previous publications that patients on paliperidone palmitate have a higher treatment continuation than patients on risperidone microspheres [19, 20, 24]. We also found that PP3M patients have a significantly lower treatment discontinuation than PP1M patients (19.2% vs 37.1%, *p*< 0.001) in the one-year follow up. The intuitively most obvious reason would be the different dosing intervals between these drugs. PP3M is to be administered every three months, whereas PP1M and risperidone microspheres are to be administered monthly and biweekly, respectively. Patients with PP3M are automatically covered for a longer duration (three months) with one injection than patients with PP1M (one month) and risperidone microspheres (two weeks). As longer treatment continuation is associated with less healthcare costs, and better quality of life by reducing relapse and hospitalizations [6–8], the findings that PP3M has superior treatment continuation could be of interest for patients and physicians. Our study focused on patients using paliperidone palmitate and risperidone microspheres, hence comparison between treatment discontinuation of paliperidone palmitate versus other LAT was not made. A previous three-year follow up study has shown that treatment discontinuation was similar among patients on PP1M or aripiprazole. On the long term, aripiprazole shows a better treatment continuation [17]. However, the study was based only on a small number (*n* = 68) of elderly Japanese patients. As studies with larger population in Europe are not available, it is not clear how PP1M compares with aripiprazole in terms of treatment continuation on the long term in Europe. As PP3M has a substantially longer dosing interval than aripiprazole and other treatment alternatives, there may be a potential advantage in reducing discontinuation. It might be worthwhile to compare treatment discontinuation between PP1M/PP3M and aripiprazole on the long term and identify which subpopulation may obtain greater benefit from PP3M in future research in Europe.

The continuation rates in this study were comparable to other studies with similar study design. A retrospective observational study using IQVIA French LRx showed non-discontinuation (for five months or longer) rate of paliperidone palmitate and risperidone microspheres was 64.5% and 35.4%, respectively [20]. In our study, the respective continuation rates were 62.5% and 32.8% by the fifth month. However, the rates were lower compared to other prospective studies. For instance, continuation rate of paliperidone palmitate (either PP1M or PP3M) was 46.2% in the one-year follow up in our study, whereas studies in UK revealed a continuation rate of 60% [25] and 65% [26]. Typically, retrospective studies demonstrate lower treatment continuation than prospective studies, because, although non-interventional, there is still the sense of the controlled trial environment, including the contact with study centres. Additionally, methodological elements influence the results. All prescription interruptions longer than the permissible gap were considered as treatment discontinuations, irrespective of reasons for the discontinuation. It is possible that in some cases the treatment is not discontinued, but the patient is, for example, hospitalized. While the above limitations are a consequence of the databases used, methodological parameters like the length of the grace period have important consequences as well. The impact of the grace period was evaluated in a sensitivity analysis using more pragmatic and longer periods. Continuation rates naturally increased with longer grace periods. As shown in our sensitivity analysis, one-year continuation rate approached results of prospective observational studies.

Treatment discontinuation is associated to disease severity, comorbidities, medication, and patient, caregiver, or physician/service factors [7]. Not surprisingly, our study shows older patients were less likely to discontinue treatment. Young patients aged 18 – 30 years may perceive themselves healthy and lack knowledge about the disease and treatment. Moreover, healthcare professionals may be reluctant to prescribe LAT to young patients due to the metabolic side effects associated with LAT [27] and the perceptions that LAI should only be used for a small subgroup of patients with chronic mental impairment (e.g. patients with frequent relapses or who pose a risk to others) [28]. Healthcare professionals should be more attentive to these young patients to improve treatment continuation. In the last decades, specialized care units for young patients have been expanding worldwide to address this issue [5, 29]. In line with previous studies [30–32], our study shows females were significantly more (5%) likely to discontinue treatment in the analysis for the primary objective. Though gender was not significant in the analysis for the secondary objective, females were still marginally more likely to discontinue treatment than males. Possible reasons could be that females usually have less severe form of schizophrenia and may be less often prescribed LATs than males [33, 34].

A qualitive study shows top reasons for treatment discontinuation were ‘positive symptoms not sufficiently improved’ and ‘medication-related adverse events’ [35]. In our study, patients on a higher dose of treatment were significantly less likely to discontinue treatment compared to patients on a relatively low dose (less than 75 mg per 30 days). It is possible that positive symptoms of patients on a low dose of treatment were not sufficiently improved. Furthermore, patients on a higher dose of treatment are likely to have a more severe form of disease and thus receive more professional and family support than those whose disease is mild. However, as data about disease severity is not available in the LRx databases, it is not possible to confirm this hypothesis. Similarly, patients on a combination therapy are more likely to experience medication-related adverse events and thus discontinue. In our study, patients on a combination therapy are 5% more likely to discontinue than patients on monotherapy. We recommend more research to study the relationship between dosage and regimen and treatment discontinuation.

In this study, patients receiving drugs prescribed by GP only were 36% more likely, whereas patients receiving drugs prescribed by psychiatrist and GP were 22% less likely to discontinue treatment compared to patients receiving drugs prescribed by psychiatrist only. This finding is consistent across the three countries. Patients are suggested to visit a psychiatrist (together or not with a GP) according to clinical practice in France and Germany but to visit a GP (together or not with a psychiatrist) in Belgium. Mental health specialists are in a key position to support improved treatment continuation through educational strategies that help patients and family members better understand and manage treatment [6, 36]. Psychiatrist and GP have complementary skills and that collaboration between GP and psychiatrist should be encouraged [37, 38]. Thus, managing patients by a multi-disciplinary team could be an effective intervention to improve treatment continuation in schizophrenia patients.

Several limitations in this study should be noted. First, the analyses performed using the LRx databases are retrospective and do not provide substantial information on patients’ clinical and functional outcomes that could be associated with treatment discontinuation. Such limitation is common to retrospective observational studies using routinely collected electronic data platforms (not designed for specific research investigations). Therefore, no conclusions can be drawn concerning possible underlying confounders such as severity of disease, prevalent complications or other individual circumstances. Second, continuation of successive transactions was taken as a proxy for treatment continuation. No data was available to show whether patients took the medications according to the prescribed treatment regimen. Rather, it was assumed that if the medication was dispensed, it was correctly administered by the patient. Third, PP3M is only indicated for patients who are adequately treated with PP1M and do not require dose adjustment. Moreover, if a patient visited a pharmacy in the panel and another out of the panel, only the first sequence of transactions was captured. The impact of these incidental off-panel visits was limited by the grace period and the sensitivity analyses. Still, treatment continuation might have been underestimated as not all patients changing pharmacy could be detected. This underestimation may be limited as chronic patients usually have high loyalty to their pharmacy.

## Conclusions

In conclusion, this study demonstrates that patients stay significantly longer on treatment when initiated on paliperidone palmitate as compared to risperidone microspheres. It also indicated a higher treatment continuation of PP3M over PP1M. Treatment continuation rates are likely to be improved by encouraging collaborative care models between psychiatrists and GPs. Further research is needed to investigate the relationship between treatment dosage, gender and age and discontinuation.

##  Supplementary Information

**Additional file 1:** **FigureS1.** Sensitivity analysis: treatmentdiscontinuation by product – patients newly initiated on paliperidone palmitateor risperidone microspheres (using a grace period of 120 days). **FigureS2.** Treatment discontinuation by product and percountry – patients newly initiated on paliperidone palmitate and risperidonemicrospheres. **Figure S3.** Treatment discontinuation by gender and per country – patientsnewly initiated on paliperidone palmitate and risperidone microspheres. **FigureS4.** Treatment discontinuation by previoustreatment and per country – patients newly initiated on paliperidone palmitateand risperidone microspheres. **FigureS5.** Treatment discontinuation by 30-day averagedose and per country – patients newly initiated on paliperidone palmitate andrisperidone microspheres. **FigureS6.** Treatment discontinuation by mono- andcombination therapy and per country – patients newly initiated on paliperidonepalmitate and risperidone microspheres. **FigureS7.** Treatment discontinuation by prescribers’specialty and per country – patients newly initiated on paliperidone palmitateand risperidone microspheres. **FigureS8.** Treatment discontinuation by age and percountry – patients newly initiated on paliperidone palmitate and risperidonemicrospheres. **FigureS9.** Sensitivity analysis: treatmentdiscontinuation by product – stable PP3M patients vs. stable PP1M patients (graceperiod: 120 days). **Table S1.** Patient characteristics of stable patients with PP1M and PP3M. **Table S2.**Univariate and multivariate Cox regression analysis of treatment discontinuationof patients with PP1M and PP3M.

## Data Availability

The data that support the findings of this study are available from IQVIA but restrictions apply to the availability of these data and so are not publicly available. Data are however available from the authors upon reasonable request and with permission of IQVIA.

## Abbreviations

APs: Antipsychotic medications
GP: General practitioner
HR: Hazard ratio
LAT: Long-acting antipsychotic treatment
LRx: Longitudinal Prescription databases
PP1M: 1-Monthly injection form of paliperidone palmitate
PP3M: 3-Monthly injection form of paliperidone palmitate
RCT: Randomized controlled trials
SmPC: Summary of product characteristics

## References

[1] Tandon R, Nasrallah HA, Keshavan MS (2009). Schizophrenia, "just the facts" 4. Clinical features and conceptualization Schizophr Res.

[2] Disease GBD, Injury I, Prevalence C (2018). Global, regional, and national incidence, prevalence, and years lived with disability for 354 diseases and injuries for 195 countries and territories, 1990–2017: a systematic analysis for the Global Burden of Disease Study 2017. Lancet.

[3] Simeone JC, Ward AJ, Rotella P, Collins J, Windisch R (2015). An evaluation of variation in published estimates of schizophrenia prevalence from 1990 horizontal line 2013: a systematic literature review. BMC Psychiatry.

[4] Lauriello J, Perkins DO. Enhancing the Treatment of Patients With Schizophrenia Through Continuous Care. J Clin Psychiatry. 2019;80(1). 10.4088/JCP.al18010ah2c 30786179

[5] Remington G, Addington D, Honer W, Ismail Z, Raedler T, Teehan M (2017). Guidelines for the Pharmacotherapy of Schizophrenia in Adults. Can J Psychiatry.

[6] Haddad PM, Brain C, Scott J (2014). Nonadherence with antipsychotic medication in schizophrenia: challenges and management strategies. Patient Relat Outcome Meas.

[7] Ascher-Svanum H, Zhu B, Faries D, Lacro JP, Dolder CR (2006). A prospective study of risk factors for nonadherence with antipsychotic medication in the treatment of schizophrenia. J Clin Psychiatry.

[8] MacEwan JP, Forma FM, Shafrin J, Hatch A, Lakdawalla DN, Lindenmayer JP (2016). Patterns of Adherence to Oral Atypical Antipsychotics Among Patients Diagnosed with Schizophrenia. J Manag Care Spec Pharm.

[9] Dilla T, Ciudad A, Alvarez M (2013). Systematic review of the economic aspects of nonadherence to antipsychotic medication in patients with schizophrenia. Patient Prefer Adherence.

[10] Thomas P (2013). Relapse: causes and consequences. Encephale.

[11] Kishimoto T, Hagi K, Nitta M, Leucht S, Olfson M, Kane JM (2018). Effectiveness of Long-Acting Injectable vs Oral Antipsychotics in Patients With Schizophrenia: A Meta-analysis of Prospective and Retrospective Cohort Studies. Schizophr Bull.

[12] Alphs L, Schooler N, Lauriello J (2014). How study designs influence comparative effectiveness outcomes: the case of oral versus long-acting injectable antipsychotic treatments for schizophrenia. Schizophr Res.

[13] Fagiolini A, Rocca P, De Giorgi S, Spina E, Amodeo G, Amore M (2017). Clinical trial methodology to assess the efficacy/effectiveness of long-acting antipsychotics: Randomized controlled trials vs naturalistic studies. Psychiatry Res.

[14] Bossie CA, Alphs LD, Correll CU (2015). Long-acting injectable versus daily oral antipsychotic treatment trials in schizophrenia: pragmatic versus explanatory study designs. Int Clin Psychopharmacol.

[15] Tiihonen J, Mittendorfer-Rutz E, Majak M, Mehtala J, Hoti F, Jedenius E (2017). Real-World Effectiveness of Antipsychotic Treatments in a Nationwide Cohort of 29823 Patients With Schizophrenia. JAMA Psychiat.

[16] Kishimoto T, Nitta M, Borenstein M, Kane JM, Correll CU (2013). Long-acting injectable versus oral antipsychotics in schizophrenia: a systematic review and meta-analysis of mirror-image studies. J Clin Psychiatry.

[17] H Suzuki, H Hibino. Comparison of treatment retention between risperidone long-acting injection, paliperidone palmitate, and aripiprazole once-monthly in elderly patients with schizophrenia. Psychogeriatrics. 2021;22(1):159–60. 10.1111/psyg.12784 34729877

[18] Taylor D, Olofinjana O (2014). Long-acting paliperidone palmitate - interim results of an observational study of its effect on hospitalization. Int Clin Psychopharmacol.

[19] Decuypere F, Sermon J, Geerts P, Denee TR, De Vos C, Malfait B (2017). Treatment continuation of four long-acting antipsychotic medications in the Netherlands and Belgium: A retrospective database study. PLoS One.

[20] Guillon P, Harmand S, Ansolabehere X (2019). Real-life persistence of long-acting injectable antipsychotics in schizophrenic patients: A retrospective observational study in France. Int J Clin Pharmacol Ther.

[21] Kap E, Kostev K (2019). The role of general practitioners and psychiatrists in issuing initiation and follow-up prescriptions for selective serotonin (norepinephrine) reuptake inhibitors in Germany. Int J Clin Pharmacol Ther.

[22] Eisen C, Lulic Z, Palacios-Moreno JM, Adalig B, Hennig M, Cortes V (2020). Persistence and adherence to dutasteride/tamsulosin fixed-dose versus free-combination alpha blocker/5ARI therapy in patients with benign prostate hyperplasia in Germany. Int J Clin Pharmacol Ther.

[23] Kap E, Konrad M, Kostev K (2019). Persistence with selective serotonin (norepinephrine) reuptake inhibitors in Germany-A retrospective database analysis. J Affect Disord.

[24] Joo SW, Shon SH, Choi G, Koh M, Cho SW, Lee J (2019). Continuation of schizophrenia treatment with three long-acting injectable antipsychotics in South Korea: A nationwide population-based study. Eur Neuropsychopharmacol.

[25] Whale R, Pereira M, Cuthbert S, Fialho R (2015). Effectiveness and Predictors of Continuation of Paliperidone Palmitate Long-Acting Injection Treatment: A 12-Month Naturalistic Cohort Study. J Clin Psychopharmacol.

[26] Attard A, Olofinjana O, Cornelius V, Curtis V, Taylor D (2014). Paliperidone palmitate long-acting injection–prospective year-long follow-up of use in clinical practice. Acta Psychiatr Scand.

[27] Sanchez-Martinez V, Romero-Rubio D, Abad-Perez MJ, Descalzo-Cabades MA, Alonso-Gutierrez S, Salazar-Fraile J (2018). Metabolic Syndrome and Cardiovascular Risk in People Treated with Long-Acting Injectable Antipsychotics. Endocr Metab Immune Disord Drug Targets.

[28] Llorca PM, Abbar M, Courtet P, Guillaume S, Lancrenon S, Samalin L (2013). Guidelines for the use and management of long-acting injectable antipsychotics in serious mental illness. BMC Psychiatry.

[29] Grover S, Avasthi A (2019). Clinical Practice Guidelines for the Management of Schizophrenia in Children and Adolescents. Indian J Psychiatry.

[30] Stentzel U, van den Berg N, Schulze LN, Schwaneberg T, Radicke F, Langosch JM (2018). Predictors of medication adherence among patients with severe psychiatric disorders: findings from the baseline assessment of a randomized controlled trial (Tecla). BMC Psychiatry.

[31] Taylor DM, Fischetti C, Sparshatt A, Thomas A, Bishara D, Cornelius V (2009). Risperidone long-acting injection: a prospective 3-year analysis of its use in clinical practice. J Clin Psychiatry.

[32] Takaesu Y, Kishimoto T, Murakoshi A, Takahashi N, Inoue Y (2016). Factors associated with discontinuation of aripiprazole treatment after switching from other antipsychotics in patients with chronic schizophrenia: A prospective observational study. Psychiatry Res.

[33] Sommer IE, Tiihonen J, van Mourik A, Tanskanen A, Taipale H (2020). The clinical course of schizophrenia in women and men-a nation-wide cohort study. NPJ Schizophr.

[34] Seeman MV (2019). Does Gender Influence Outcome in Schizophrenia?. Psychiatr Q.

[35] Ascher-Svanum H, Nyhuis AW, Stauffer V, Kinon BJ, Faries DE, Phillips GA (2010). Reasons for discontinuation and continuation of antipsychotics in the treatment of schizophrenia from patient and clinician perspectives. Curr Med Res Opin.

[36] Kirk Morton N, Zubek D (2013). Adherence challenges and long-acting injectable antipsychotic treatment in patients with schizophrenia. J Psychosoc Nurs Ment Health Serv.

[37] Fredheim T, Danbolt LJ, Haavet OR, Kjonsberg K, Lien L (2011). Collaboration between general practitioners and mental health care professionals: a qualitative study. Int J Ment Health Syst.

[38] Reilly S, Planner C, Gask L, Hann M, Knowles S, Druss B, et al. Collaborative care approaches for people with severe mental illness. Cochrane Database Syst Rev. 2013(11):CD009531. 10.1002/14651858.CD009531.pub2 24190251

