# Supplementary material for: Assessment of risk factors of treatment discontinuation among patients on paliperidone palmitate and risperidone microspheres in France, Germany and Belgium
Source: BMC Psychiatry. 2022 Jun 7;22:382. doi: 10.1186/s12888-022-03914-2 (PMC9171957; doi:10.1186/s12888-022-03914-2)

Title: Assessment of risk factors of treatment discontinuation among patients on paliperidone palmitate and risperidone microspheres in France, Germany and Belgium

## Table S1. Patient characteristics of stable patients with PP1M and PP3M

|  |  | **Total** | **France** | **Germany** | **Belgium** |
| --- | --- | --- | --- | --- | --- |
| *Total* |  | 19,693 (100%) | 8,057 (100%) | 10,236 (100%) | 1,400 (100%) |
| *Gender* |  |  |  |  |  |
|  | Women | 7,364 (37%) | 2,700 (34%) | 4,143 (40%) | 521 (37%) |
|  | Men | 12,329 (63%) | 5,357 (66%) | 6,093 (60%) | 879 (63%) |
| *Age* |  |  |  |  |  |
|  | 18-30 years | 3,319 (17%) | 1,678 (21%) | 1,641 (16%) | - |
|  | 31-50 years | 9,269 (47%) | 4,319 (54%) | 4,950 (48%) | - |
|  | >50 years | 5,705 (29%) | 2,060 (26%) | 3,645 (36%) | - |
| *Type of treatment* | |  |  |  |  |
|  | PP1M | 15,771 (80%) | 6,503 (81%) | 8,121 (79%) | 1,147 (82%) |
|  | PP3M | 3,922 (20%) | 1,554 (19%) | 2,115 (21%) | 253 (18%) |
| *Previous treatment* | |  |  |  |  |
|  | Other LAT | 19,693 (100%) | 8,057 (100%) | 10,236 (100%) | 1,400 (100%) |
| *Average dosage per 30 days^*^* | |  |  |  |  |
|  | <75 mg | 3,736 (19%) | 1,450 (18%) | 1,937 (19%) | 349 (25%) |
|  | 75-125 mg | 8,806 (45%) | 3,114 (39%) | 5,123 (50%) | 569 (41%) |
|  | >125 mg | 7,151 (36%) | 3,493 (43%) | 3,176 (31%) | 482 (34%) |
| *Combination therapy* | |  |  |  |  |
|  | Monotherapy | 11,259 (57%) | 5,114 (63%) | 5,421 (53%) | 724 (52%) |
|  | Combination therapy | 8,434 (43%) | 2,943 (37%) | 4,815 (47%) | 676 (48%) |
| *Specialty of the prescribers* | |  |  |  |  |
|  | GP only | 657 (3%) | 275 (3%) | 259 (3%) | 123 (9%) |
|  | Psychiatrist + GP | 2,577 (13%) | 951 (12%) | 872 (9%) | 754 (54%) |
|  | Psychiatrist only | 15,343 (78%) | 6,348 (79%) | 8,621 (84%) | 374 (27%) |
|  | Other | 1,116 (6%) | 483 (6%) | 484 (5%) | 149 (11%) |
| *Other treatments^^^* | |  |  |  |  |
|  | None | 5,895 (30%) | 1,451 (18%) | 4,148 (41%) | 296 (21%) |
|  | 1 molecule | 2,947 (15%) | 1,102 (14%) | 1,619 (16%) | 226 (16%) |
|  | 2 molecules | 2,458 (12%) | 1,027 (13%) | 1,224 (12%) | 207 (15%) |
|  | >2 molecules | 8,393 (43%) | 4,477 (56%) | 3,245 (32%) | 671 (48%) |

***** The recommended monthly dose of the three study drugs was similar per SmPC (50 mg per 30 days), i.e. risperidone microspheres for 25 mg every two weeks, PP1M for 50 mg per month and PP3M for 175 mg per three months.

^ Based on the number of distinct molecules other than antipsychotics purchased, patients were classified into four groups: patients who purchased 0, 1, 2 and more than 2 distinct other molecules during the first three months after index.

## Table S2. Univariate and multivariate Cox regression analysis of treatment discontinuation of patients with PP1M and PP3M

|  |  |  |  | **Univariate** | |  | **Multivariate (including Belgian data)** | |  | **Multivariate (excluding Belgian data)** | |
| --- | --- | --- | --- | --- | --- | --- | --- | --- | --- | --- | --- |
| Variable | Reference |  |  | HR | P value^ |  | HR | P value^ |  | HR | P value^ |
| Gender | Men | Women |  | 1.02 | 0.19 |  | 1.01 | 0.77 |  | 1.03 | 0.071 |
| Age* | 18-30 years | 31-50 years |  | 0.86 | **<.0001** |  | - | **-** |  | 0.87 | **<.0001** |
|  |  | >50 years |  | 0.82 | **<.0001** |  | - | **-** |  | 0.82 | **<.0001** |
| Type of treatment | PP3M | PP1M |  | 1.35 | **<.0001** |  | 1.33 | **<.0001** |  | 1.32 | **<.0001** |
| Dosage per 30 days | <75 mg | 75-125 mg |  | 0.95 | **0.020** |  | 0.95 | **0.016** |  | 0.96 | 0.070 |
|  |  | >125 mg |  | 0.84 | **<.0001** |  | 0.85 | **<.0001** |  | 0.85 | **<.0001** |
| Combination therapy | Monotherapy | Combination therapy |  | 1.07 | **<.0001** |  | 1.10 | **<.0001** |  | 1.09 | **<.0001** |
| Specialty of the prescribers | Psychiatrist only | GP only |  | 1.08 | 0.086 |  | 1.04 | 0.41 |  | 1.07 | 0.16 |
|  |  | Psychiatrist + GP |  | 0.93 | **0.003** |  | 0.90 | **<.0001** |  | 0.88 | **<.0001** |
|  |  | Other |  | 0.94 | 0.086 |  | 0.91 | **0.007** |  | 0.92 | **0.023** |
| Other treatments^#^ | None | 1 molecule |  | 0.98 | 0.54 |  | - | - |  | - | - |
|  |  | 2 molecules |  | 0.97 | 0.22 |  | - | - |  | - | - |
|  |  | >2 molecules |  | 1.00 | 0.85 |  | - | - |  | - | - |

*Age was not available in the Belgian database. The Belgian data was not included in the univariate analysis using age as a covariate but was used all remaining univariate analyses.

^P-values in bold are p-values < 0.05.

# Other treatment was not included in multivariate analysis as the p-value > 0.20 in the univariate analysis.

## Figure S1. Sensitivity analysis: treatment discontinuation by product – patients newly initiated on paliperidone palmitate or risperidone microspheres (using a grace period of 120 days)


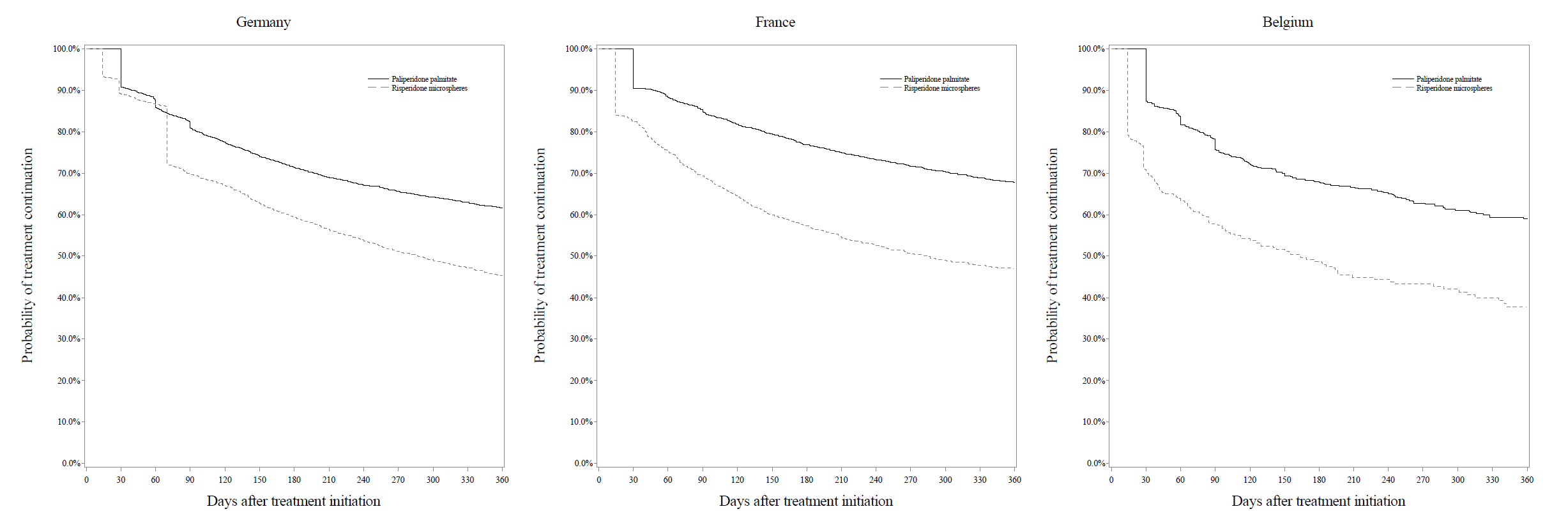


## Figure S2. Treatment discontinuation by product and per country – patients newly initiated on paliperidone palmitate and risperidone microspheres


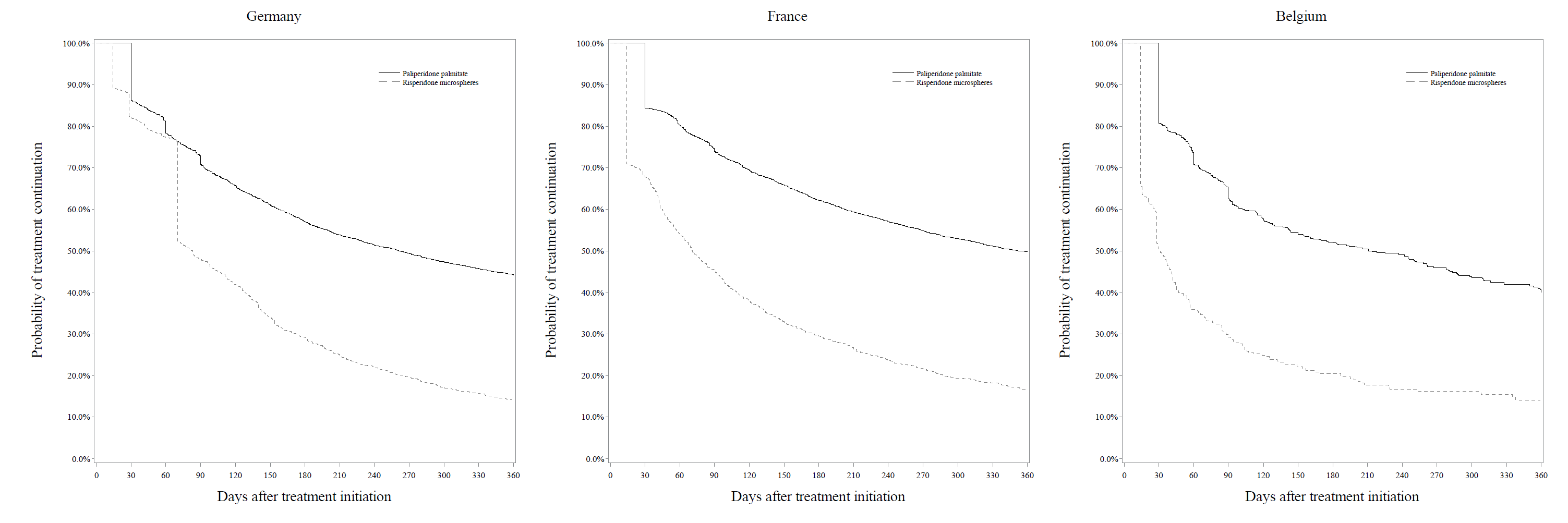


## Figure S3. Treatment discontinuation by gender and per country – patients newly initiated on paliperidone palmitate and risperidone microspheres


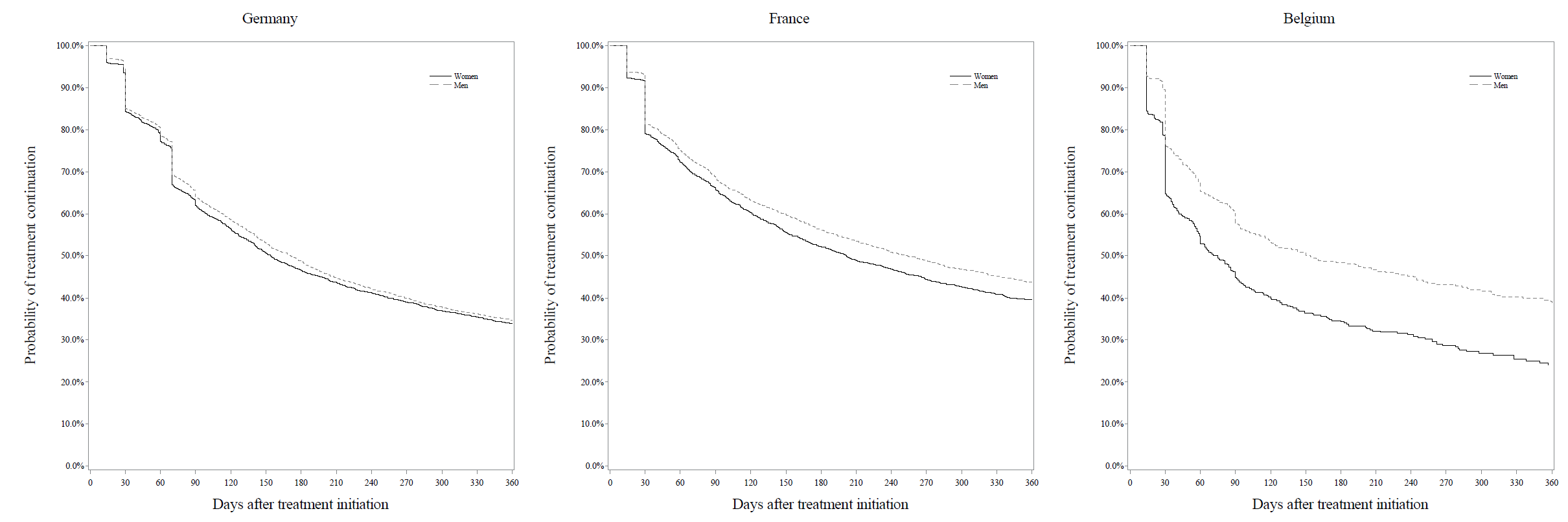


## Figure S4. Treatment discontinuation by previous treatment and per country – patients newly initiated on paliperidone palmitate and risperidone microspheres


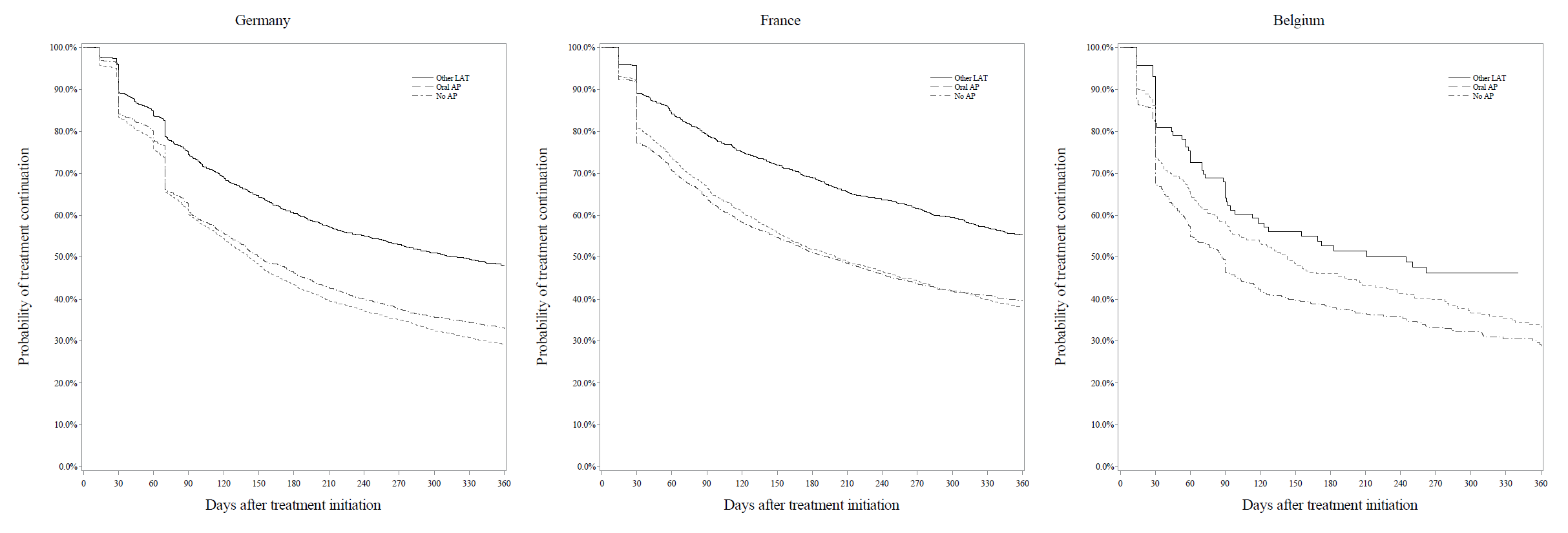


## Figure S5. Treatment discontinuation by 30-day average dose and per country – patients newly initiated on paliperidone palmitate and risperidone microspheres


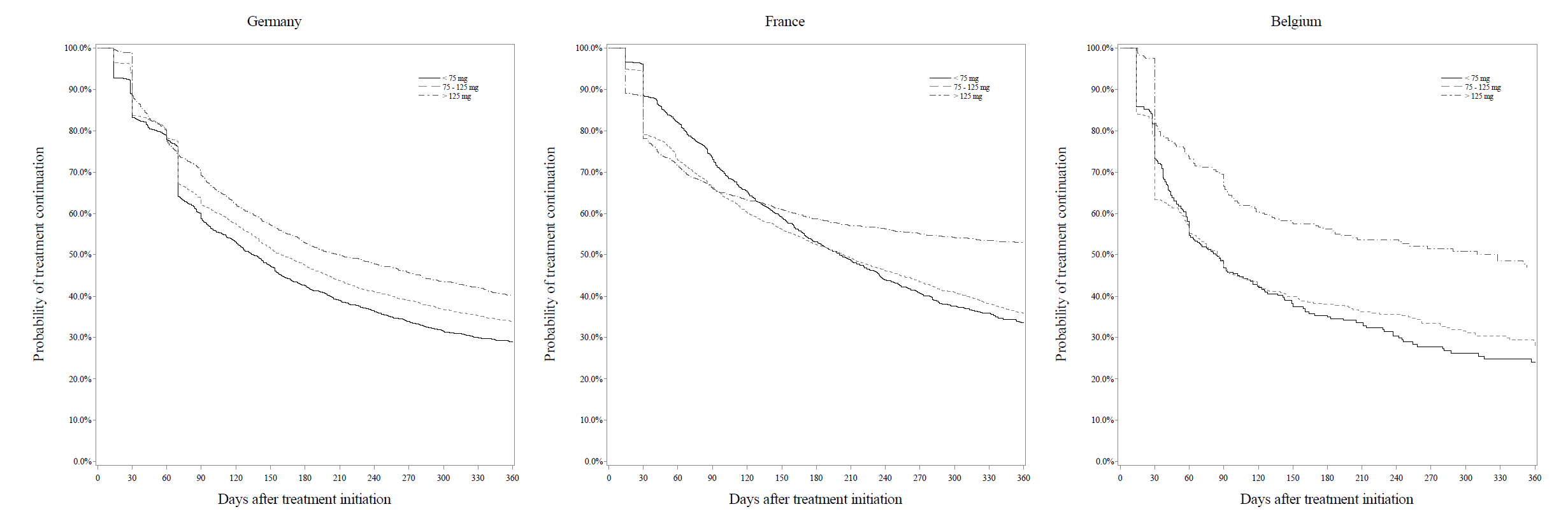


The recommended monthly dose of the three study drugs was similar per SmPC (50 mg per 30 days), i.e. risperidone microspheres for 25 mg every two weeks, PP1M for 50 mg per month and PP3M for 175 mg per three months.

## Figure S6. Treatment discontinuation by mono- and combination therapy and per country – patients newly initiated on paliperidone palmitate and risperidone microspheres


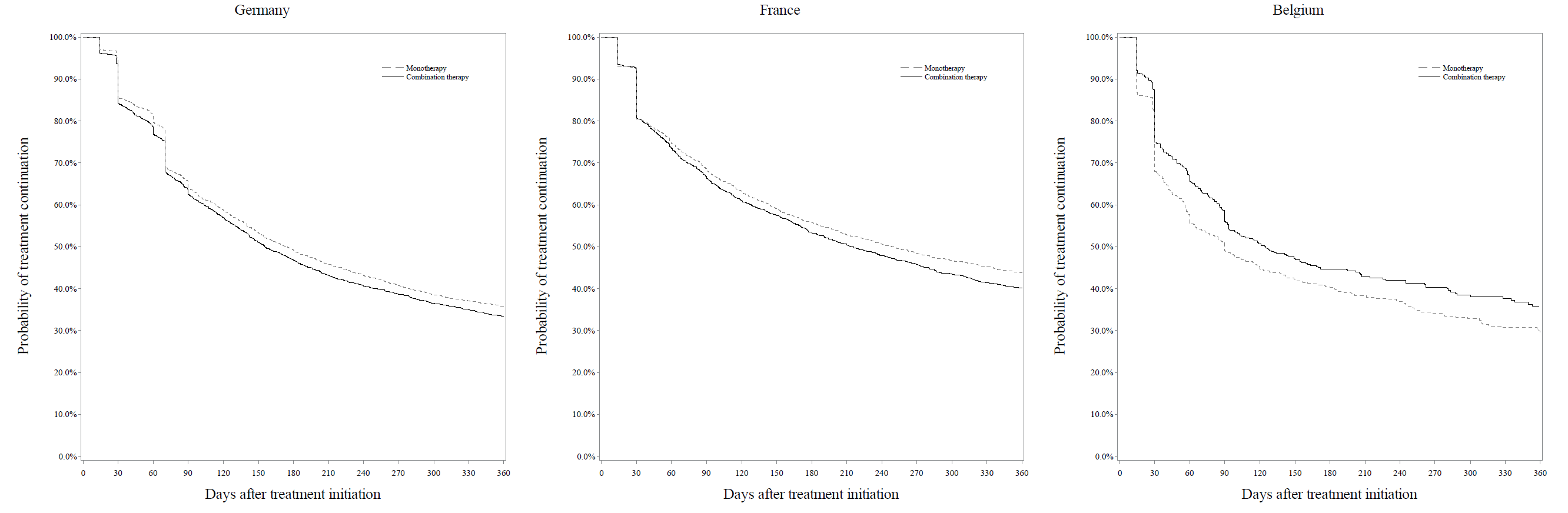


## Figure S7. Treatment discontinuation by prescribers’ specialty and per country – patients newly initiated on paliperidone palmitate and risperidone microspheres


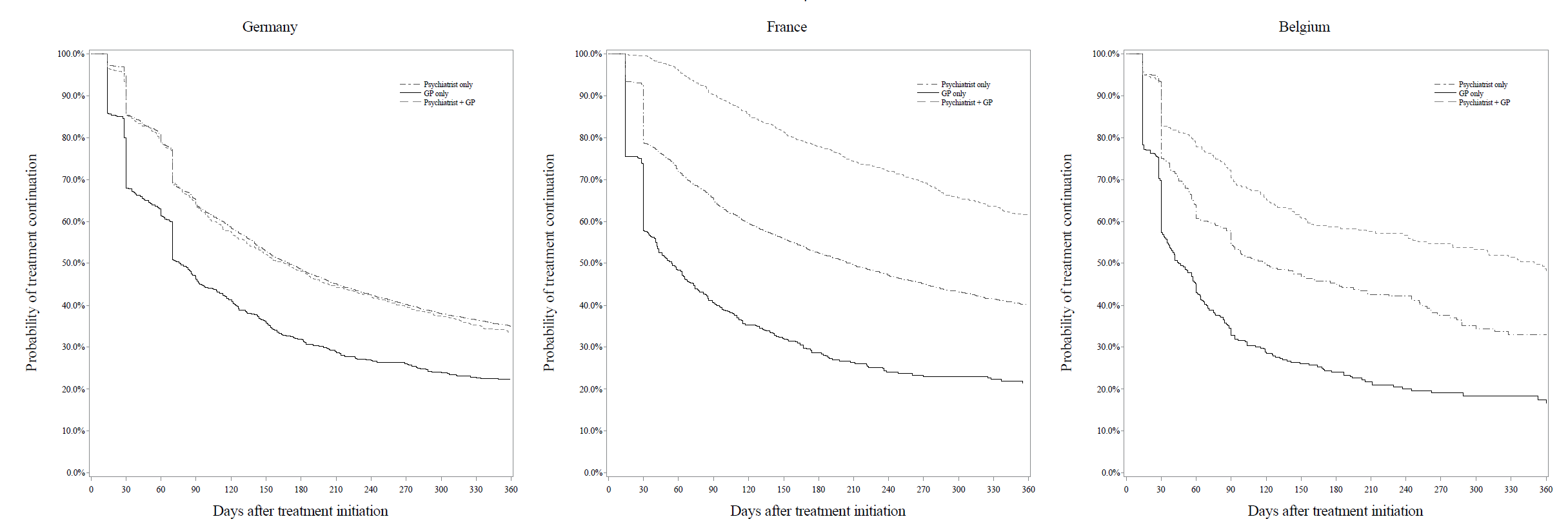


## Figure S8. Treatment discontinuation by age and per country – patients newly initiated on paliperidone palmitate and risperidone microspheres


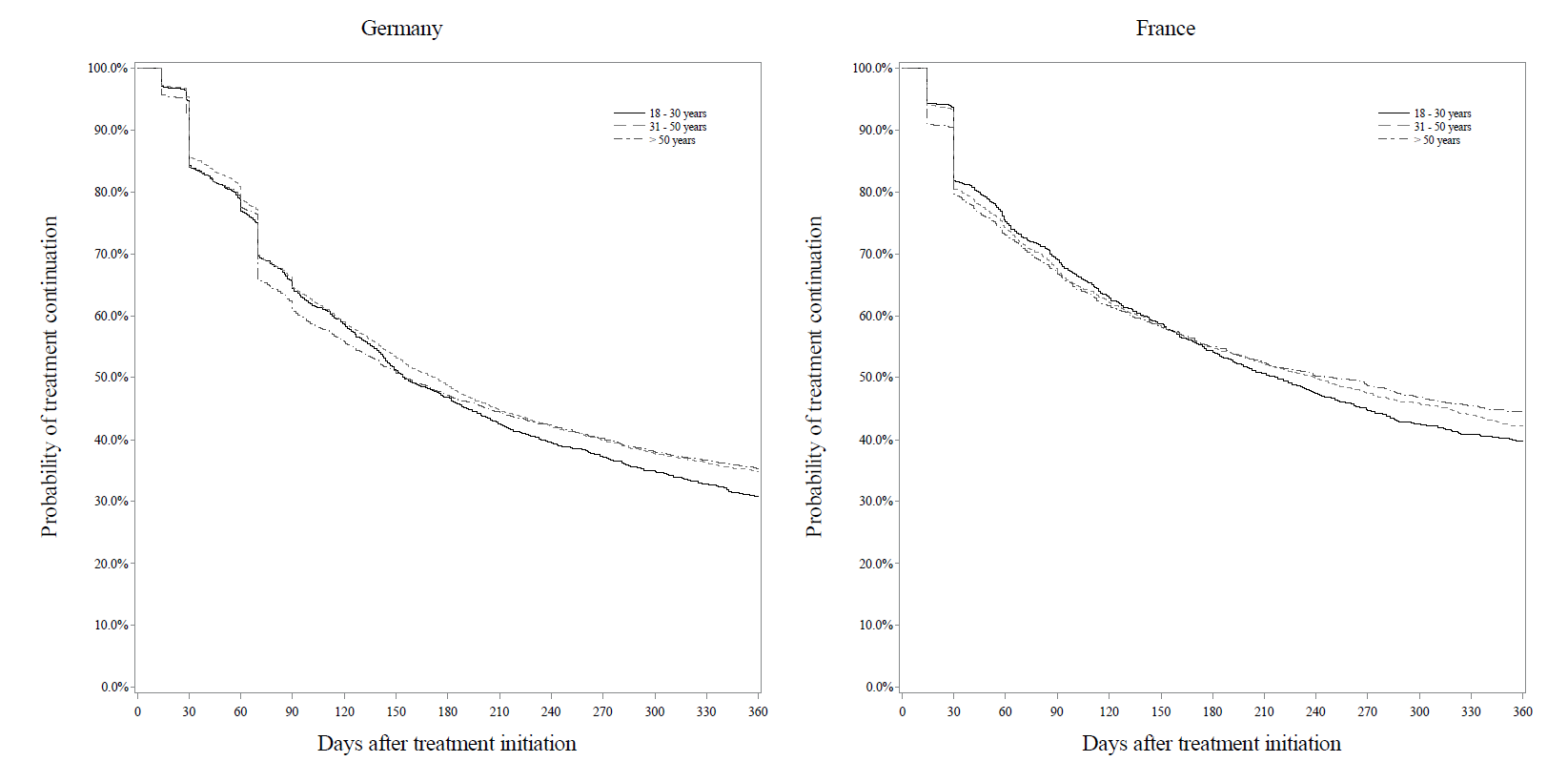


## Figure S9. Sensitivity analysis: treatment discontinuation by product – stable PP3M patients vs. stable PP1M patients (grace period: 120 days)


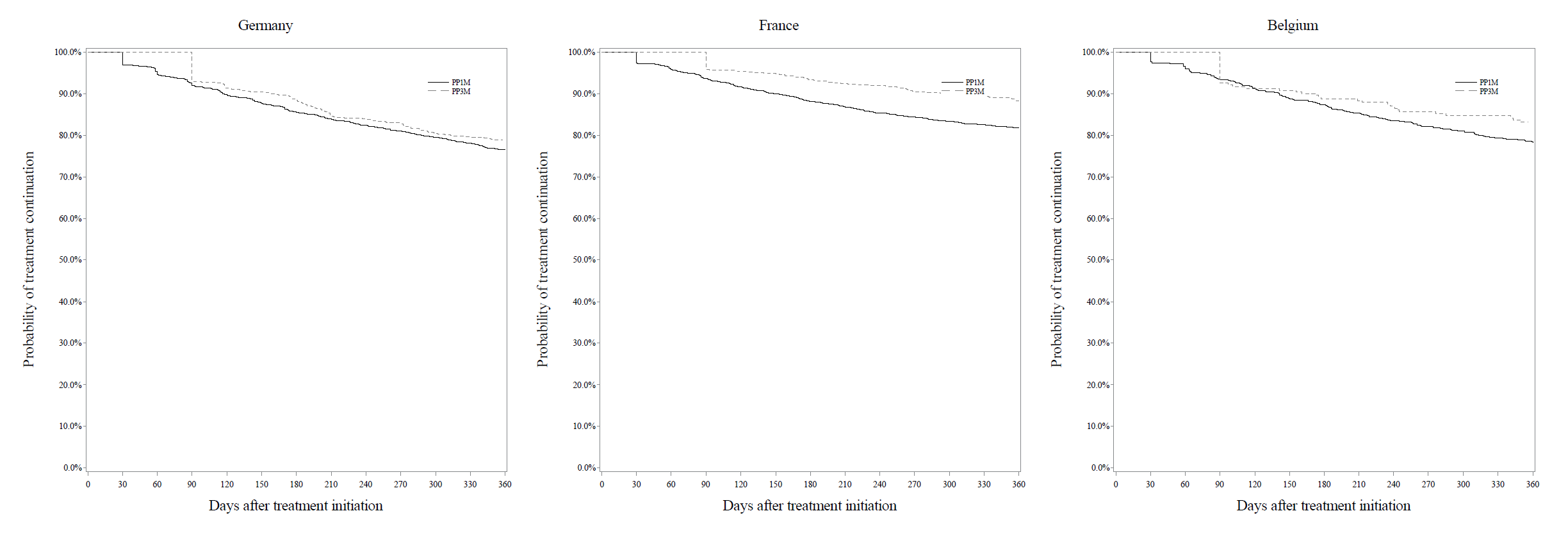

Supplement: Supplementary file 1 — Additional file 1: FigureS1. Sensitivity analysis: treatmentdiscontinuation by product – patients newly initiated on paliperidone palmitateor risperidone microspheres (using a grace period of 120 days). FigureS2. Treatment discontinuation by product and percountry – patients newly initiated on paliperidone palmitate and risperidonemicrospheres. Figure S3. Treatment discontinuation by gender and per country – patientsnewly initiated on paliperidone palmitate and risperidone microspheres. FigureS4. Treatment discontinuation by previoustreatment and per country – patients newly initiated on paliperidone palmitateand risperidone microspheres. FigureS5. Treatment discontinuation by 30-day averagedose and per country – patients newly initiated on paliperidone palmitate andrisperidone microspheres. FigureS6. Treatment discontinuation by mono- andcombination therapy and per country – patients newly initiated on paliperidonepalmitate and risperidone microspheres. FigureS7. Treatment discontinuation by prescribers’specialty and per country – patients newly initiated on paliperidone palmitateand risperidone microspheres. FigureS8. Treatment discontinuation by age and percountry – patients newly initiated on paliperidone palmitate and risperidonemicrospheres. FigureS9. Sensitivity analysis: treatmentdiscontinuation by product – stable PP3M patients vs. stable PP1M patients (graceperiod: 120 days). Table S1. Patient characteristics of stable patients with PP1M and PP3M. Table S2.Univariate and multivariate Cox regression analysis of treatment discontinuationof patients with PP1M and PP3M. [file 12888_2022_3914_MOESM1_ESM.docx]
